# Outcomes of short- versus long-acting gas tamponades in vitrectomy for rhegmatogenous retinal detachment

**DOI:** 10.1186/s40942-024-00530-y

**Published:** 2024-02-05

**Authors:** Verena Schöneberger, Jeany Q. Li, Leonie Menghesha, Frank G. Holz, Friederike Schaub, Tim U. Krohne

**Affiliations:** 1 Department of Ophthalmology, University Medical Center Rostock, University of Rostock, Doberaner Str. 140, 18057 Rostock, Germany; 2 Department of Ophthalmology, Faculty of Medicine and University Hospital Cologne, University of Cologne, Cologne, Germany; 3 Department of Ophthalmology, University of Bonn, Bonn, Germany

**Keywords:** Gas tamponade, Vitrectomy, Retinal detachment, Surgery, Sulfur hexafluoride (SF6), Hexafluoroethane (C2F6), Octafluoropropane (C3F8)

## Abstract

**Background:**

In vitrectomy for rhegmatogenous retinal detachment, long-acting gas tamponades (LGT) such as C3F8 or C2F6 may improve surgical success rate due to their prolonged effect compared to a short-acting gas tamponade (SGT) with SF6. On the other hand, SGT allow a significantly faster visual rehabilitation after surgery and may reduce the risk of gas-related complications. As comparative data in retinal detachment surgery is limited, we assessed the outcomes of vitrectomies using either LGT or SGT.

**Methods:**

We retrospectively analyzed 533 eyes of 524 consecutive patients diagnosed with primary rhegmatogenous retinal detachment not complicated by proliferative vitreoretinopathy (PVR) and treated by vitrectomy at two clinical sites. Depending on the site the patients presented at, they received either preferentially LGT (study site 1) or SGT (study site 2). Retinal re-detachment rates during a period of 6 months following surgery were analyzed.

**Results:**

At study site 1, 254 of 278 eyes (91.4%) were treated by LGT (C3F8 72.3%; C2F6 19.1%), whereas at study site 2, 246 of 255 eyes (96.5%) received SGT (SF6). Rates of retinal re-detachment in the LGT- and SGT-treated groups were similar with 23 of 254 eyes (9.1%) and 24 of 246 eyes (9.8%), respectively (*p* = 0.9). Median time to re-detachment was 5.7 weeks in the LGT-treated group and 4.4 weeks in the SGT-treated group (*p* = 0.4).

**Conclusion:**

In rhegmatogenous retinal detachment repair by vitrectomy, the use of SGT results in comparable rates of successful retinal re-attachment as LGT. Given the faster visual rehabilitation with SGT, these results suggest SGT as a sensible alternative to LGT in surgery of retinal detachment without PVR.

## Background

In surgical repair of rhegmatogenous retinal detachment, vitrectomy with coagulation of the retinal break and subsequent installation of a gas tamponade is today the most commonly employed technique [1]. Both short-acting gas tamponades such as sulfur hexafluoride (SF6) and long-acting gas tamponades with perfluorinated short-chain carbon compounds such as hexafluoroethane (C2F6) or octafluoropropane (C3F8) are widely used. As C2F6 is not approved by the US Food and Drug Administration (FDA), SF6 and C3F8 are more commonly used in the US [2]. SF6 is mostly used in a non-expansile concentration of 20% in air, C2F6 at a non-expansile concentration of 16% and C3F8 14% with approximately maximal expansion after 21, 27, and 30 h after injection, respectively [3, 4]. The longer the carbon chain, the lower the water solubility and the longer the intraocular longevity [5]. Resorption times of these gases in the eye differ considerably with approximately 10–14 days, 5–6 weeks, and 8–10 weeks for SF6, C2F6, and C3F8, respectively [4, 6, 7]. 

As presence of an intraocular gas tamponade impairs vision significantly, a short-acting gas tamponade allows for faster visual rehabilitation of the patient following surgery, thus improving patient comfort. Also, shorter duration of the gas tamponade may reduce the risk of postoperative gas-related complications such as intraocular pressure decompensation and cataract formation [8]. On the other hand, long-acting gas tamponades allow for a longer support of the retinal break following coagulation and may thus result in improved sealing of the break with a lower rate of retinal re-detachment [9]. Thus, the use of short-acting gas tamponades may offer several advantage including increased patient comfort but would only be justified if it did not result in an increased re-detachment rate.

In the current literature, air tamponades have been compared with different gas tamponades in rhegmatogenous retinal detachment with regard to re-detachment rate, with some studies reporting higher surgical success rates with gas tamponades and others finding no difference [1, 10, 11]. However, available comparative data on long- and short-acting gas tamponades in standard retinal detachment surgery is sparse. In high-risk situations such as retinal detachment in highly myopic eyes or retinal detachment complicated by proliferative vitreoretinopathy (PVR), studies have compared silicone oil to gas tamponade. A meta-analysis showed no major differences in outcomes between C3F8 and silicone oil, but silicone oil outperformed SF6 in eyes with PVR, [12] and further studies have described higher single surgery success rates with C3F8 compared to SF6 [13, 14]. 

As comparative data for different gas tamponades such as C3F8, C2F6, and SF6 in uncomplicated rhegmatogenous retinal detachment is limited, we analyzed single surgery success rates of vitrectomy in eyes with retinal detachment using either long- or short acting gas tamponades.

## Methods

We retrospectively analyzed the medical charts of consecutive patients that had undergone vitrectomy for primary rhegmatogenous retinal detachment at two clinical sites (Department of Ophthalmology, University of Bonn, Germany; Department of Ophthalmology, University of Cologne, Germany). Due to different departmental preferences, treatment at study site 1 was predominantly performed using long-acting gas tamponades of either 14% C3F8 or 16% C2F6 in air, whereas study site 2 predominantly employed short-acting gas tamponades with 20% SF6 in air. Otherwise, surgical technique was comparable at both sites, i.e.three-port pars plana vitrectomy was performed, usually 23 gauge, less commonly 20 gauge or 25 gauge. A complete vitrectomy was performed including indentation of the periphery. Posterior vitreous detachment was induced if not already present. Heavy liquid for temporary attachment during surgery and drainage of subretinal fluid was performed at the individual decision of the surgeon. Retinal breaks or degenerations have been identified and were treated usually by cryopexy, alternatively by laserpexy. A silicone oil tamponade was employed only in selected cases such as inferior breaks and inability of the patient to comply with posturing, and these cases were excluded from the comparison of short- versus long-acting gas tamponades. All surgeries were performed by one of two experienced vitreoretinal surgeons (FS, TUK) with one of them operating at both sites.

Exclusion criteria with regard to the study eye were previous retinal detachment, previous vitrectomy, PVR grade B or C, degenerative myopia, long-standing atrophic retinal detachment, retinal detachment secondary to retinoschisis, giant retinal tear, proliferative diabetic retinopathy, and previous perforating eye injury.

Patients were quasi-randomized in the two study groups ‘C3F8/C2F6’ or ‘SF6’ solely based on their decision to which of the two nearby study site they initially presented at. Specifically, all consecutive patients presenting at study site 1 and treated using C3F8 or C2F6 were included in study group ‘C3F8/C2F6’ whereas all consecutive patients presenting at study site 2 and treated using SF6 were included in study group ‘SF6’.

A postoperative examination was usually performed at the study site 6 weeks after surgery, while the subsequent follow-up examinations were conducted by the referring ophthalmologist. Patient charts were retrospectively analyzed for a review period of 6 months after surgery. Patients that did present again with a retinal re-detachment were considered treatment failures whereas the others were considered treatment successes.

In descriptive statistics, means with standard deviations were used for variables with presumed Gaussian distribution (e.g. age) and medians with interquartile ranges were used for variables without (e.g. time to re-detachment). Statistical comparisons were performed using Fisher’s exact test for two-by-two contingency tables (e.g. re-detachment rates) or Mann-Whitney test for nonparametric, independent variables (i.e. time to re-detachment) as indicated (Prism 8, GraphPad, San Diego, USA). Differences with a two-tailed p value of 0.05 or less were considered statistically significant.

## Results

A total of 533 eyes of 524 consecutive patients were recruited for the study (Table 1). Group 1 includes 278 eyes of 272 patients. Mean age in this group was 60.2 years (± 10.2), and 59.0% were male. A majority of 254 eyes (91.4%) received a long-acting gas tamponade, including C3F8 in 201 eyes (72.3%) and C2F6 in 53 eyes (19.1%). A SF6 tamponade was applied in 6 eyes (2.2%) and a silicone oil tamponade in 18 eyes (6.5%).

Group 2 comprises 255 eyes of 252 patients with a mean age of 63.7 years (± 10.1) and 66.5% male. In group 2, 246 eyes (96.5%) received a short-acting gas tamponade with SF6, 1 eye (0.4%) a long-acting gas tamponade with C3F8, and 8 eyes (3.1%) a silicone oil tamponade. In both study sites 1 and 2, a majority of 89.9% and 94.5%, respectively, of eyes were operated by small gauge (usually 23-gauge, less commonly 25-gauge) vitrectomy with the remainder being 20-gauge vitrectomies.

**Table 1:** Baseline patient characteristics

	Study site 1	Study site 2
n [eyes (patients)]	278 (272)	255 (252)
Age [years; mean (± SD)]	60.2 (± 10.2)	61.1 (± 14.2)
Sex [male; %]	59.0	67.1
Vitrectomy		
23/25-gauge [eyes (%)]	250 (89.9)	241 (94.5)
20-gauge [eyes (%)]	28 (10.1)	14 (5.5)
Endotamponade		
C3F8 [eyes (%)]	201 (72.3)	1 (0.4)
C2F6 [eyes (%)]	53 (19.1)	0 (0)
C3F8 or C2F6 [eyes (%)]	254 (91.4)	1 (0.4)
SF6 [eyes (%)]	6 (2.2)	246 (96.5)
Silicone oil [eyes (%)]	18 (6.5)	8 (3.1)

Due to the retrospective nature of the study, standardized assessment of further baseline parameters such as extend of retinal detachment, macular status, and location of retinal break were not available for all eyes and these parameters were therefore not analyzed.

**Fig. 1:**
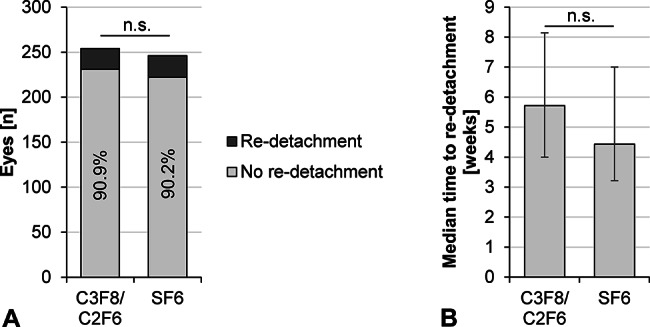
Comparison of surgical outcomes between C3F8- or C2F6-treated eyes (*n* = 254) and SF6-treated eyes (*n* = 246). (**A**) The rate of retinal re-detachment after a follow-up period of 6 months was not statistically different (not significant, n.s.) between the groups (*p* = 0.9; Mann-Whitney test). (**B**) In eyes with retinal re-detachment, the time between surgery and re-detachment (median with interquartile range) was not statistically different between groups (*p* = 0.4; Fisher’s exact test)

Within the postoperative review time of 6 months, re-detachment rates in study site 1 and 2 were 9.0% and 9.4% overall and did not exhibit a statistical difference (*p* = 0.9). Similarly, in the subgroups of C3F8/C2F6-treated eyes of group 1 (*n* = 254) and SF6-treated eyes of group 2 (*n* = 246), re-detachment rates were similar with 9.1% and 9.4%, respectively (*p* = 0.9; Fisher’s exact test; Fig. 1). The median time between surgery and re-detachment was longer in group 1 with 5.7 weeks (interquartile range, IQR, 4.0-8.1) compared to group 2 with 4.4 weeks (IQR, 3.2-7.0) but without statistically significant difference (*p* = 0.4; Mann-Whitney test).

Power analysis demonstrated the sample size to be sufficient to detect a difference of at least 6.6% between study groups and 7.2% between subgroups (power 0.8, significance level 0.05).

## Discussion

SF6, C2F6, and C3F8 all represent well established tamponade agents for the treatment of retinal detachments with vitrectomy. Comparative data on the effect of the different gas tamponades on the success rate in retinal detachment surgery are sparse. Thus, the selection of the specific gas is a decision the surgeon tends to base on experience and subjective criteria [6]. 

Advantages of short-acting gas tamponade for the patient include a faster visual rehabilitation following surgery. Due to the large difference in refractive index between the injected gas and the posterior surface of the lens, all gas tamponades induce a temporary myopic shift of about 8 to 50 diopters that precludes sharp vision until gas re

sorption [15]. As the resorption time of SF6 is significantly shorter compared to long-acting gas tamponades, SF6 has the obvious advantage of allowing a faster visual recovery following surgery, thus increasing patient comfort. Moreover, SF6 as compared to C3F8 has been shown to result in a decreased incidence of gas-related postoperative complications such as cataract formation and intraocular pressure rise [8]. Cataracts following gas tamponade can occur as both transient posterior subcapsular cataract or progressive nuclear sclerosis, with the latter often requiring additional surgery [16, 17]. 

Despite these advantages of short-acting gas tamponades, their shorter tamponade effect may potentially result in a higher retinal re-detachment rate compared to long-acting gas tamponades. The questions whether SF6 is inferior to C2F6 and C3F8 with regard to primary success rate in retinal detachment surgery has been unresolved so far and was, therefore, subject of this study. Herein, we measured surgical success by means of absence of retinal re-detachment during a postoperative review period of 6 months in 533 eyes treated at two clinical sites. Selection of the gas tamponade was based on the site the patient presented to, with one site using near-exclusively long- and the other near-exclusively short-acting gas tamponades. All other aspects of treatment were comparable between both sites, including the preferential employment of small-gauge vitrectomy with cryopexy of the retinal break and postoperative positioning of the patient. All surgeries were performed by either of two experienced vitreoretinal surgeons, with one of them operating at both sites.

Our results demonstrate a similar re-detachment rate of 9.1% (23 of 254 eyes) in the C3F8/C2F6-treated and of 9.8% (24 of 246 eyes) in the SF6-treated group. Median time to re-detachment was 5.7 weeks in the C3F8/C2F6-treated and 4.4 weeks in the SF6-treated group. Both surgical outcomes were not statistically different between the groups. Our results are in line with the literature, such as a current meta-analysis by Chen et al. comparing gas tamponades with air tamponade. Ten studies with 2,677 eyes were included, of which 1,556 eyes received different types of gas tamponades and the remainder an air tamponade. The reported primary anatomical success rates with gas tamponades range from 78.1 to 100% with different gas tamponades, [11] similar to our study. The lowest rate was in a prospective study with C3F8 gas tamponade in inferior breaks, with a success rate of 78.13% in 32 eyes [18]. The largest study was conducted by Govers et al., reporting retinal re-detachment in 11 of 458 eyes of a mixed group treated with either SF6 or C3F8 and a 2.4% re-detachment rate [1]. Similarly, other studies describe anatomical outcomes in mixed SF6 and C3F8/C2F6 cohort but do not compare SF6 with C3F8/C2F6, e.g. a 6.5% re-detachment rate in a mixed SF6 and C2F6 cohort, [19] and a 4% re-detachment rate in a mixed SF6, C2F6 and C3F8 cohort with only macula-on RRD cases [20]. 

This study provides, to the best of our knowledge, the first comparison of SF6 and C3F8/C2F6 gas tamponades in standard retinal detachment surgery. Strengths of the study are the large number of 533 included eyes and their quasi-randomization into the two treatment groups solely based on which of two nearby study site the patients presented at and, thus, selection bias secondary to surgeon’s choice of gas tamponade was limited. Limitations of the study include the retrospective nature of our analysis, the associated lack of a standardized assessments of patient parameters at baseline, and the resulting limited availability of some functional and anatomical baseline data.

## Conclusion

In rhegmatogenous retinal detachment repair by vitrectomy, the use of LGT and SGT results in comparable rates of successful retinal re-attachment. Our study demonstrates a very similar primary surgical success rate of about 91% with both long-acting gas tamponades (C3F8 or C2F6) and short-acting gas tamponade (SF6). Thus, our data suggests SF6 to be as effective as C3F8 or C2F6 in surgical treatment of standard retinal detachment not complicated by e.g. PVR or high myopia. Given the advantage of SF6 in terms of increased patient comfort due to faster postoperative visual recovery and its potential advantages with regard to gas-related complications such as cataract and intraocular pressure rise, our findings suggest SF6 as a sensible alternative to long-acting gas tamponades in rhegmatogenous retinal detachment repair.

## Data Availability

All data generated or analyzed during this study are included in this article. Further enquiries can be directed to the corresponding author.

## Abbreviations

C2F6: hexafluoroethane
C3F8: octafluoropropane
FDA: Food and Drug Administration
IQR: interquartile range
LGT: long-acting gas tamponade
PVR: proliferative vitreoretinopathy
SF6: sulfur hexafluoride
SGT: short-acting gas tamponade
